# Interleukin-34 produced by human fibroblast-like synovial cells in rheumatoid arthritis supports osteoclastogenesis

**DOI:** 10.1186/ar3693

**Published:** 2012-01-20

**Authors:** Seung-Jun Hwang, Bongkun Choi, Soon-Suk Kang, Jae-Ho Chang, Yong-Gil Kim, Yeon-Ho Chung, Dong Hyun Sohn, Min Wook So, Chang-Keun Lee, William H Robinson, Eun-Ju Chang

**Affiliations:** 1 Department of Anatomy and Cell Biology, University of Ulsan College of Medicine, Seoul 138-736, Korea; 2 Department of Medicine, Graduate School, Cellular Dysfunction Research Center and BMIT, University of Ulsan College of Medicine, Seoul 138-736, Korea; 3 Department of Natural Science, College of Natural Science, Sang-Ji University, Wonju 220-702, Korea; 4 Department of Internal Medicine, Division of Rheumatology, University of Ulsan College of Medicine, Seoul 138-736, Korea; 5 Department of Medicine, Division of Immunology and Rheumatology, Stanford University School of Medicine, Stanford CA 94305, USA

## Abstract

**Introduction:**

Interleukin-34 (IL-34) is a recently defined cytokine, showing a functional overlap with macrophage colony stimulating factor (M-CSF). This study was undertaken to address the expression of IL-34 in rheumatoid arthritis (RA) patients and to investigate its regulation and pathogenic role in RA.

**Methods:**

IL-34 levels were determined in the RA synovium, synovial fluid (SF) and fibroblast-like synovial cells (FLS) by immunohistochemistry, real-time PCR, enzyme-linked immunosorbent assay and immunoblotting. RA activity was assessed using Disease Activity Score 28 (DAS28) activity in the plasma collected at baseline and one year after treatment. Conditioned media (CM) were prepared from RA FLS culture with tumor necrosis factor alpha (TNFα) for 24 hours and used for functional assay.

**Results:**

IL-34 was expressed in the synovium, SF, and FLS from RA patients. The production of IL-34 in FLS was up-regulated by TNFα in RA samples compared with osteoarthritis (OA) patients. Importantly, the preferential induction of IL-34 rather than M-CSF by TNFα in RAFLS was mediated by the transcription factor nuclear factor kappa B (NF-κB) and activation of c-Jun N-terminal kinase (JNK). IL-34 elevation in plasma from RA patients was decreased after the administration of disease-modifying anti-rheumatic drugs (DMARDs) in accordance with a decrease in DAS28. CM from RAFLS cultured with TNFα promoted chemotactic migration of human peripheral blood mononuclear cells (PBMCs) and subsequent osteoclast (OC) formation, effects that were attenuated by an anti-IL-34 antibody.

**Conclusions:**

These data provide novel information about the production of IL-34 in RA FLS and indicate that IL-34 is an additional osteoclastogenic factor regulated by TNFα in RA, suggesting a discrete role of IL-34 in inflammatory RA diseases.

## Introduction

Rheumatoid arthritis (RA) is an autoimmune chronic inflammatory disease characterized by bone and cartilage destruction that is mediated by bone-resorbing osteoclasts (OCs) [1,2]. OCs differentiate from the monocyte/macrophage lineage of hematopoietic myeloid progenitors in response to macrophage colony-stimulating factor (M-CSF) and RANKL (receptor activator of NF-κB ligand) [3,4] and participate in a variety of inflammatory bone degenerative diseases. OC differentiation correlates with the severity of the inflammatory condition [2]. OCs mediate erosive bone resorption at the bone-pannus interface of the synovium in RA joints resulting from chronic inflammation of multiple synovial joints [5]. Synovial fluid (SF) produced by the inflamed synovium in joints, hyperplasic synovial fibroblasts, and activated synovial T cells increases the production of RANKL and several inflammatory cytokines [6,7]. These inflammatory conditions lead to enhanced OC formation and the subsequent increase in resorbing activity [2].

Tumor necrosis factor alpha (TNFα) is well established as a critical OC differentiation-enhancing factor that acts by mediating mobilization of osteoclast precursors (OCPs) from bone marrow into the inflamed joint, where they appear to contribute to inflammatory erosive arthritis [8]. TNFα-stimulated fibroblast-like synovial cells (FLS) increase cytokine production [6], which accelerates OC formation in the inflamed synovium of RA [9]. Thus, the administration of TNFα blocking agents results in a decrease in the pathological changes indicative of RA inflammatory responses, and as such provides a potential clinical benefit [10]. Recent studies have shown that administration of an antibody (Ab) against the M-CSF receptor, c-Fms or inhibitor, selectively and completely blocks osteoclastogenesis and bone erosion induced by TNFα injection or inflammatory arthritis [11,12], suggesting a link between TNFα and c-Fms under pathological inflammatory conditions. Accordingly, identifying factors involved in TNFα-induced OCPs mobilization and subsequent differentiation that contribute to erosive arthritis is a matter of considerable interest.

The recently discovered cytokine IL-34 binds to the M-CSF receptor c-Fms [13]. The functional similarity of IL-34 and M-CSF is demonstrated by their role in osteoclastogenesis [14-18]. Although IL-34 and M-CSF share the c-Fms receptor, their signal transduction mechanisms and biological activity are not identical [16]. Functional overlap [16], but differential expression, of M-CSF and IL-34 has been observed in the context of M-CSF receptor-mediated regulation of myeloid cells [18]. However, whether IL-34 is involved in RA pathogenesis is still unknown.

## Materials and methods

### Patients and reagents

All RA patients enrolled in this study fulfilled the 1987 revised criteria of the American College of Rheumatology [19]. Patients were compared with age- and sex-matched control patients with OA.

Informed consent was obtained from all patients and the experimental protocol was approved by the Human Research Ethics Committee of the University of Ulsan College of Medicine (2010-871). SF was collected from the knee joint (with or without swelling) of each patient by sterile aspiration and centrifuged at 250*g *for 10 minutes. Cell-free RA synovial fluid (RA SF) was stored at -70°C until used. Synovia were isolated from seven patients with RA (mean age, 59.9 ± 12.3; range, 42 to 75 years) and seven patients with OA (mean age, 62.5 ± 8.2; range, 46 to 77 years). All patients were undergoing knee replacement surgery. For the one-year follow-up study, sera were collected from the patients listed in Additional file 1 at baseline and one year after treatment from ten patients with RA. RA activity was simultaneously assessed using Disease Activity Score 28 (DAS28) activity [20].

Pyrrolidine dithiocarbamate (PDTC), SP600125, SB203580, PD98059, and anti-β-actin antibody was purchased from Sigma (St. Louis, MO, USA). Rabbit anti-human IL-34 for immunoblotting was from ProSci Incorporated (Poway, CA, USA). Mouse anti-human IL-34 for blocking IL-34 activity, mouse polyclonal immunoglobulin G (IgG), IL-17, TNFα, and IL-1β were purchased from R&D Systems (Minneapolis, MN, USA). Recombinant M-CSF, RANKL, and IL-34 were purchased from PeproTech, Inc. (Rocky Hill, NJ, USA).

### Immunohistochemistry

OA and RA synovia for immunohistochemistry analysis were collected in accordance with experimental protocols approved by the Stanford University Institutional Review Board and with the patients' informed consent as described [12]. Sections of optimal cutting temperature (OCT) compound-embedded synovial tissue samples were cut on a cryostat (Thermo Scientific, Kalamazoo, MI, USA) and fixed in 4% paraformaldehyde for 10 minutes at 4°C. The slides were then washed twice (5 minutes each) with PBS and permeabilized with 0.1% Triton X-100 for 3 minutes at room temperature. After washing with PBS, endogenous peroxidase activity was quenched with 0.03% H_2_O_2 _solution. The slides were blocked with 7.5% normal goat serum for 20 minutes and thereafter incubated with 4 μg/ml of IL-34 antibody (LSBio, Seattle, WA, USA) or control rabbit IgG (Abcam, Cambridge, MA, USA) for 20 minutes at room temperature. Following two washes with PBS, the slides were incubated with 1:200 diluted biotinylated secondary antibody (Vectastain EliteABC Kit; Vector Laboratories, Orton Southgate, Peterborough, UK) for 30 minutes. After washing with PBS, the slides were incubated with complexed avidin-biotin horseradish peroxidase ABC reagent (Vectastain Elite ABCkit; Vector) for 30 minutes, and then exposed to 3,3-diaminobenzidine (DAB) substrate according to the manufacturer's instructions (Vector). The nuclei were subsequently counterstained with hematoxylin (Vector). Tissues were examined and photographed with an Olympus BX51 microscope outfitted with an Olympus DP72 digital camera (Olympus).

### Isolation of FLS and OCPs from human peripheral blood mononuclear cells (PBMCs)

FLS were isolated by enzymatic digestion of synovial tissues obtained from RA and OA patients undergoing total joint replacement surgery, as previously described [21]. Blood was collected from healthy volunteers and PBMCs were obtained from whole blood by centrifugation over Ficoll gradients (Pharmacia Biotech, Uppsala, Sweden) at 400*g *for 30 minutes and erythrocytes were removed by lysing in ACK buffer (0.15 M NH_4_Cl, 1 mM KHCO_3 _and 0.1 mM EDTA, pH 7.2).

### Conventional reverse transcriptase-polymerase chain reaction (RT-PCR) PCR and quantitative real-time RT-PCR

Total RNA was extracted from synovial tissues obtained from three RA patients, as indicated, using the RNeasy Mini Kit (QIAGEN, Hilden, Germany). cDNA was synthesized from total RNA using murine Moloney leukemia virus reverse transcriptase (MMTV-RT, Gibco BRL, Gaithersburg, MD, USA). The cDNA was amplified using the Takara PCR amplification kit (Takara Biotechnology, Shiga, Japan); PCR was carried out in a Perkin Elmer thermal cycler. Quantitative RT-PCR (qRT-PCR) was performed using Power SYBR Green 1-Step Kit and an ABI 7000 Real Time PCR System (Applied Biosystems, Carlsbad, CA, USA) according to the manufacturer's instructions. The sequences of primers used were as follows: IL-34, 5'-TAC AGG AGC CGA CTT CAG-3' (sense) and 5'-CTC ACC AAG ACC CAC AGA-3' (antisense); M-CSF, 5'-GCT GCT TCA CCA AGG ATT ATG-3' (sense) and 5'- GGG TCA CTG CTA GGG ATG-3' (antisense); and GAPDH (glyceraldehyde 3-phosphate dehydrogenase), 5'-AGC CAC ATC GCT CAG ACA-3' (sense) and 5'-GCC CAA TAC GAC CAA ATC C-3' (antisense). Results are expressed as the ratio of target PCR product relative to GAPDH product. The amplification protocol consisted of an initial reverse transcription step at 48°C for 30 minutes, followed by 40 cycles of denaturation at 95°C for 15 seconds and annealing and extension at 60°C for 1 minute.

### Enzyme-linked immunosorbent assay (ELISA)

IL-34 concentration was measured using an IL-34-specific sandwich ELISA (R&D systems, Minneapolis, MN, USA) in accordance with the protocols of the manufacturer. Recombinant human IL-34 serially diluted in culture media was used as a standard. ELISA plates were incubated with mouse anti-human IL-34 Ab and then blocked with 3% BSA in PBS for 1 hour at room temperature. SF from patients with RA and OA, or supernatants from FLS cultures were added to the plates. After washing with PBS containing 0.05% Tween 20, each well was incubated with biotinylated mouse anti-human IL-34 (0.5 μg/ml) for 2 hours at room temperature. Plates were then washed with PBS containing 0.05% Tween 20 and incubated with streptavidin conjugated to horseradish peroxidase (HRP) for 1 hour at room temperature. A color reaction was developed with tetramethylbenzidine solution, and then stopped with 0.1 M H2SO4. The absorbance at 450 nm was measured using a Bio-Rad microtiter plate reader (Bio-Rad Laboratories, Hercules, CA, USA).

### Immunoblotting

RA FLS were collected and proteins were resolved by sodium dodecyl sulfate-polyacrylamide gel electrophoresis and transferred to nitrocellulose paper (GE Healthcare, Piscataway, NJ, USA). The membranes were immunoblotted with an anti-human IL-34 rabbit polyclonal IgG or β-actin antibody. Bound antibodies were visualized with HRP-conjugated antibodies against rabbit or mouse IgG using an enhanced chemiluminescence Western blotting system (Millipore, Billerica, MA, USA).

### PBMCs migration assay

Transmigration assays were performed as follows using PBMCs: conditioned media (CM) was prepared by collecting supernatant from RA FLS cultured in the presence of TNFα (10 ng/ml) for 24 hours. CM from RA FLS cultures, recombinant IL-34 (100 ng/ml), or CM plus anti-IL-34 Ab was added to the lower compartments of a 5-μm pore Transwell system (Costar, Corning, NY, USA). PBMCs (1.2 × 10^6^) were added to the upper chamber of each transwell and allowed to migrate for 6 hours. The number of PBMCs in the lower chamber was then counted.

### OC differentiation

For osteoclastogenesis, PBMCs were plated on 48-well plates and cultured in α-MEM supplemented with 10% fetal bovine serum (FBS) and cultured with RANKL (100 ng/ml) plus M-CSF (100 ng/ml) as a control as described [21] or RANKL (100 ng/ml) plus IL-34 (100 ng/ml). RANKL was added at a final concentration of 100 ng/ml to CM obtained from RA FLS cultured in the presence of TNFα (10 ng/ml) for 24 hours. Osteoclastic differentiation was maintained for 12 to 14 days prior to tartrate resistant acid phosphatase (TRAP) staining and scoring of multinucleated OCs, as previously described [22].

### Statistical analysis

Means ± standard deviations (SDs) were calculated for all conditions, and differences between means were analyzed using Student's *t*-test. A *P*-value < 0.05 was considered significant. Patient data were tested by the Mann-Whitnney U test.

## Results

### High levels of IL-34 in synovia and SF from patients with RA

**Figure 1:**
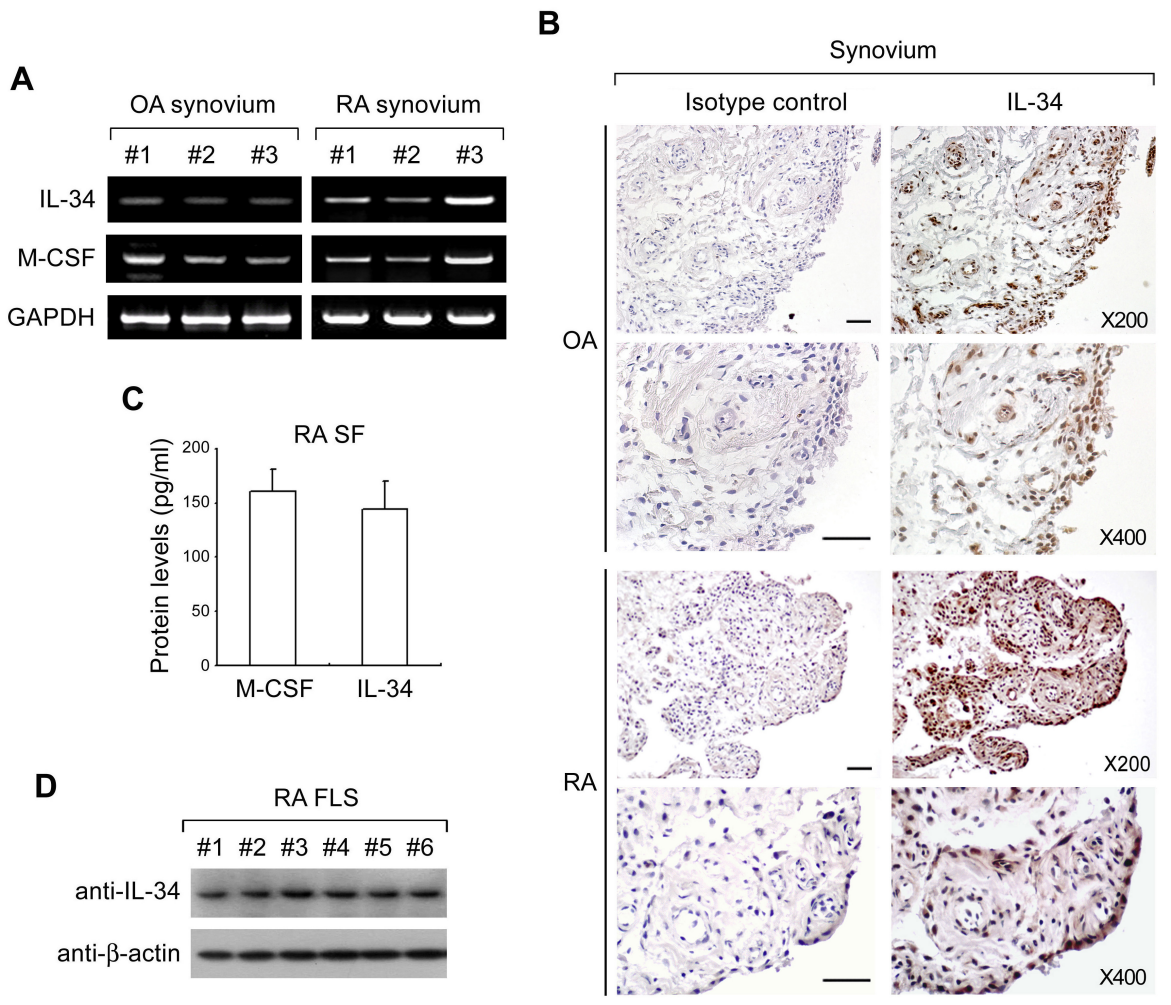
**Expression of interleukin-34 (IL-34) in human rheumatoid arthritis (RA) synovium and synovial fluid (SF)**. (**A**) IL-34 and macrophage colony-stimulating factor (M-CSF) mRNA levels in synovia from patients with RA (*n *= 3) and osteoarthritis (OA) (*n *= 3) were determined by reverse transcriptase PCR (RT-PCR). Synovium were homogenized and lysed, and total RNA was extracted as described in Materials and Methods. Glyceraldehyde 3-phosphate dehydrogenase (GAPDH) mRNA levels were detected as a control. (**B**) Representative immunohistochemical images of OA or RA synovia stained with antibodies against IL-34 or isotype controls. Images are shown at 200× (upper) and 400× (lower) magnification. Scale bars = 100 μm. (**C**) Synovial fluid (SF) from RA patients (*n *= 7) (RA SF) was collected and the concentrations of M-CSF and IL-34 were measured using an enzyme-linked immunosorbent assay (ELISA) assay. (**D**) Expression of IL-34 protein in fibroblast-like synovial cells (FLS) from RA patients (*n *= 6) was determined by immunoblotting against human IL-34. Whole-cell lysates of RA FLS cells were resolved by SDS-PAGE and followed by immunoblotting with anti-human IL-34 and anti-β-actin antibodies.

To demonstrate the involvement of IL-34 in RA pathogenesis, we first determined whether synovia and SF from patients with RA (RA SF) displayed high levels of IL-34. IL-34 mRNA expression was detected in synovia obtained from three RA patients at levels similar to those of M-CSF (Figure 1A); high levels of IL-34 mRNA (Figure 1A) and protein (Figure 1B) were observed in human RA synovium compared to OA synovium. The levels of IL-34 protein in RA SF were comparable to those of M-CSF (Figure 1C). In addition, we found that IL-34 protein was expressed in FLS from RA patients (Figure 1D). These observations indicate that IL-34 is expressed in synovial tissue, synovial fluid, and FLS from RA patients, suggesting that IL-34 is an additional factor involved in the pathogenesis of RA.

### TNFα-induced IL-34 production by FLS of RA patients

**Figure 2:**
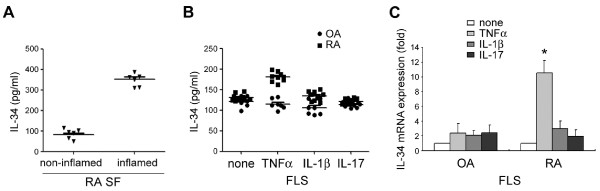
**Effect of tumor necrosis factor alpha (TNFα) on the expression of interleukin-34 (IL-34) in rheumatoid arthritis (RA) and osteoarthritis (OA) fibroblast-like synovial cells (FLS)**. (**A**) Paired samples of non-inflamed (*n = *7) and inflamed (*n *= 7) synovial fluid (SF) from individual RA patients were collected and analyzed for IL-34 by an enzyme-linked immunosorbent assay (ELISA). Closed triangles indicate individual data points; horizontal bars show group means (*P *< 0.01, inflamed versus non-inflamed RA SF samples). (**B**) Fibroblast-like synovial cells (FLS) were prepared and cultured as described in Materials and Methods. RA FLS (*n *= 9) and OA FLS (*n *= 7) were cultured with different cytokines (10 ng/ml of TNFα, IL-1β, or IL-17) for 24 hours, and the concentration of IL-34 in the media was measured by ELISA. Closed squares or circles indicate individual RA or OA data points, respectively; horizontal bars show group means (*P *< 0.01, IL-34 concentration in RA versus OA after TNFα treatment). (**C**) OA and RA FLS were treated with different cytokines (10 ng/ml of TNFα, IL-1β, or IL-17) for 24 hours. IL-34 mRNA levels in cell lysates were determined by quantitative reverse transcriptase-PCR (qRT-PCR). The relative expression of IL-34 mRNA was obtained using the cycle threshold (Ct) method after normalization to glyceraldehyde 3-phosphate dehydrogenase (GAPDH). Data are presented as fold-change of gene expression compared to untreated controls. Bars indicate the mean and SD of IL-34 mRNA expression relative to that of GAPDH (*P < 0.05, N.S., not significant, compared to none treated).

FLS act as the main cell mediating the cytokine-rich environment of the inflamed synovium in RA pathogenesis [23] and stimulate OC differentiation and activity in the inflammatory bone destruction by regulating RANKL expression [24,25]. Interestingly, we found that the level of soluble IL-34 in RA SF from inflamed RA synovium (351.7 ± 30.7 pg/ml) with joint swelling was significantly higher than that in RA SF from non-inflamed RA synovium (81.1 ± 24.5 pg/ml; *P *< 0.01) (Figure 2A). We therefore determined whether RA FLS produced IL-34 in response to pro-inflammatory cytokines. RA FLS (*n *= 9) or OA FLS (*n *= 4), used as a disease control, were cultured with TNFα, IL-1β, or IL-17 (each at 10 ng/ml) for 24 hours. Interestingly, the increase in IL-34 concentration was more marked in TNFα-stimulated RA FLS than in IL-1β- or IL-17-stimulated RA FLS (Figure 2B). In contrast, OA FLS showed no significant changes in those responses (Figure 2B). Moreover, an analysis of IL-34 mRNA levels using quantitative real-time RT-PCR showed that IL-34 mRNA expression was markedly increased in TNFα-stimulated RA FLS compared to controls (*P *< 0.01) (Figure 2C). IL-1β and IL-17 treatment induced a smaller increase in IL-34 mRNA expression (Figure 2C). However, the level of IL-34 expression in control OA FLS was not significantly modulated by these cytokines (Figure 2C). These results provide the first demonstration that RA FLS produce IL-34 and that this production is enhanced by TNFα.

### Differential induction of IL-34 by RA FLS treated with TNFα

**Figure 3:**
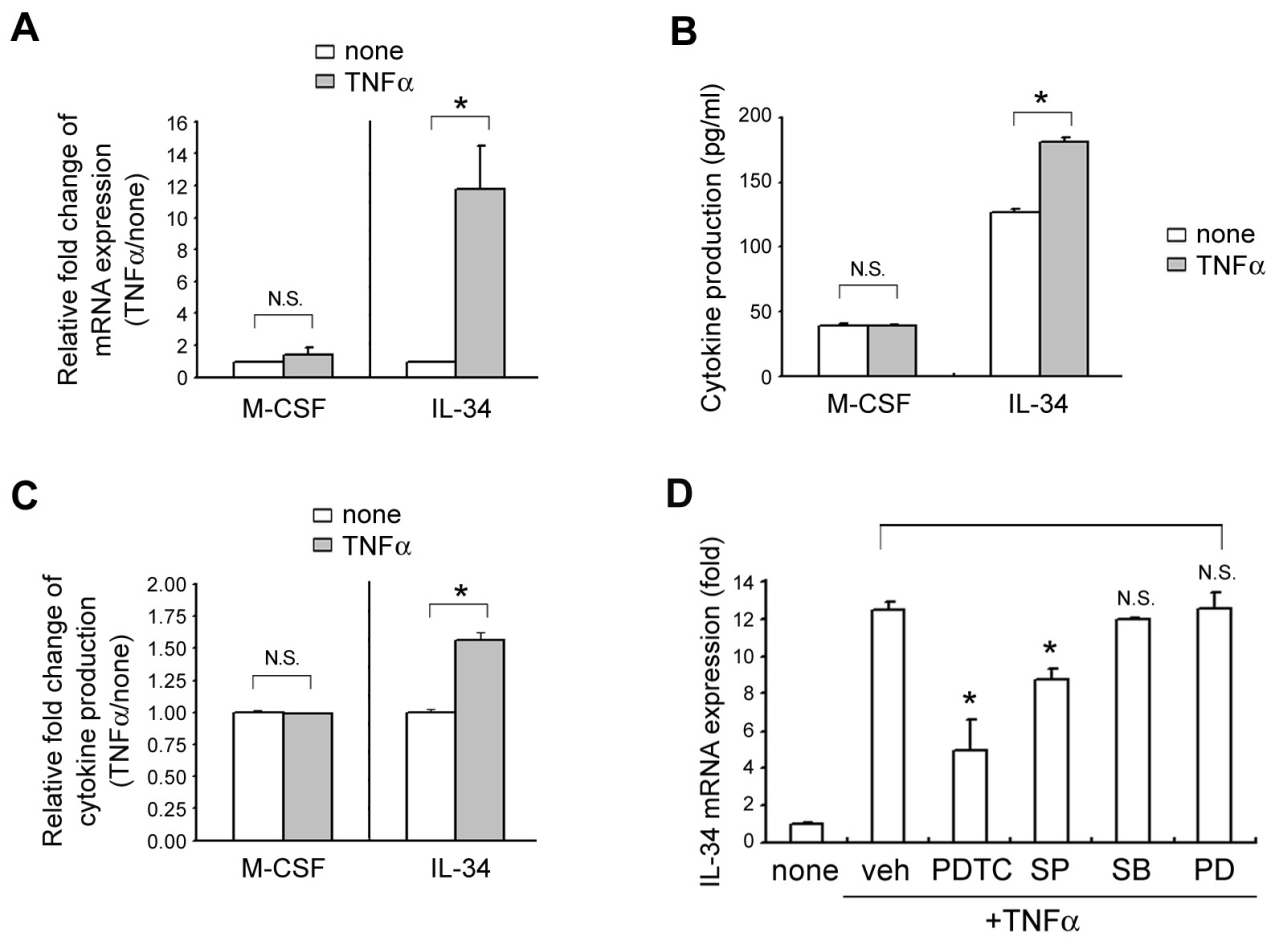
**Effect of tumor necrosis factor alpha (TNFα) on the expression of interleukin-34 (IL-34) and macrophage colony-stimulating factor (M-CSF) in rheumatoid arthritis (RA) fibroblast-like synovial cells (FLS)**. (**A**) RA FLS were treated with or without 10 ng/ml TNFα for 24 hours, and M-CSF and IL-34 mRNA levels were determined in cell lysates by quantitative reverse transcriptase-PCR (qRT-PCR). The relative expression of M-CSF (left) and IL-34 (right) mRNA after TNFα treatment is presented as fold-change in gene expression compared to untreated controls (none). Bars indicate the mean and SD of M-CSF or IL-34 mRNA expression relative to that of glyceraldehyde 3-phosphate dehydrogenase (GAPDH) (*P< 0.05, N.S., not significant, compared to none). (**B**) Cells were cultured as described in panel A, and the concentration of M-CSF (left) or IL-34 (right) in the medium was measured by an enzyme-linked immunosorbent assay (ELISA). (**C**) TNFα-mediated induction of M-CSF (left) or IL-34 (right) protein production was converted into fold change relative to that of control (none). Bars indicate the mean and SD of M-CSF or IL-34 protein relative to that of control (*P < 0.05, N.S., not significant, compared to none). (**D**) Nuclear factor kappa B (NF-κB) and c-Jun N-terminal kinase (JNK) dependent IL-34 mRNA expression upon TNFα stimulation in RA FLS. RA FLS were pretreated with the vehicle (dimethyl sulfoxide, 10 μM), NF-κB inhibitor, pyrrolidine dithiocarbamate(10 μM), the JNK inhibitor, SP600125 (10 μM), the p38 inhibitor, SB203580 (10 μM), or the extracellular signal-regulated protein kinase (ERK) inhibitor, PD98059 (10 μM), for 30 minutes and then treated with 10 ng/ml TNFα for 24 hours. Untreated RA FLS were used as a control (none). M-CSF and IL-34 mRNA levels were determined in cell lysates by qRT-PCR. The relative expression of IL-34 mRNA is presented as fold change in gene expression compared to untreated vehicle controls (*P < 0.05, N.S., not significant, compared to veh). none, no treatment; veh, vehicle; SP, SP600125; SB, SB203580; PD, PD98059.

IL-34 and M-CSF were recently reported to share the same receptor, c-Fms [13]. Our data showed that IL-34 expression was enhanced after treatment with TNFα Figure 2), suggesting that differential production of IL-34, regulated by the pathological condition of RA, can replace M-CSF function. To test this possibility, we next examined whether TNFα differentially regulates IL-34 and M-CSF expression. RA FLS constitutively expressed and produced both M-CSF and IL-34 (Figure 3A). TNFα treatment caused a 12-fold increase in IL-34 mRNA expression above this basal level (Figure 3A, right), but had little effect on M-CSF mRNA expression (Figure 3A, left). Together, the level of IL-34 protein in RA FLS was much greater than M-CSF production in the presence of TNFα (Figure 3B), showing approximately a 1.5-fold increase in TNFα-treated RA FLS over that of the control RA FLS (Figure 3C). Thus, these data demonstrate the differential regulation of IL-34 and M-CSF expression by TNFα.

TNFα activates NF-κB and JNK, which are important for TNFα-mediated gene expression and are involved in the activation of FLS [26]. To understand better the mechanism by which TNFα increases IL-34 production in RA FLS, we examined the effects of inhibitors of NF-κB or JNK on IL-34 expression. RA FLS were pretreated with the vehicle, NF-κB inhibitor, PDTC, or the JNK inhibitor, SP600125, at a concentration of 10 μM for 30 minutes followed by incubation with TNFα for 24 hours. As shown in Figure 3D, the NF-κB inhibitor, PDTC, effectively reduced TNFα-induced IL-34 mRNA expression; moreover, JNK inhibition modestly decreased the TNFα-induced elevation of IL-34. However, p38 or extracellular signal-regulated protein kinase (ERK) inhibition did not affect TNFα-induced IL-34 mRNA expression (Figure 3D). Collectively, these results suggest that the TNFα-induced increase in IL-34 expression in RA FLS is mediated by NF-κB and JNK pathways.

### Clinical determination of IL-34 in plasma from patients with RA

**Figure 4:**
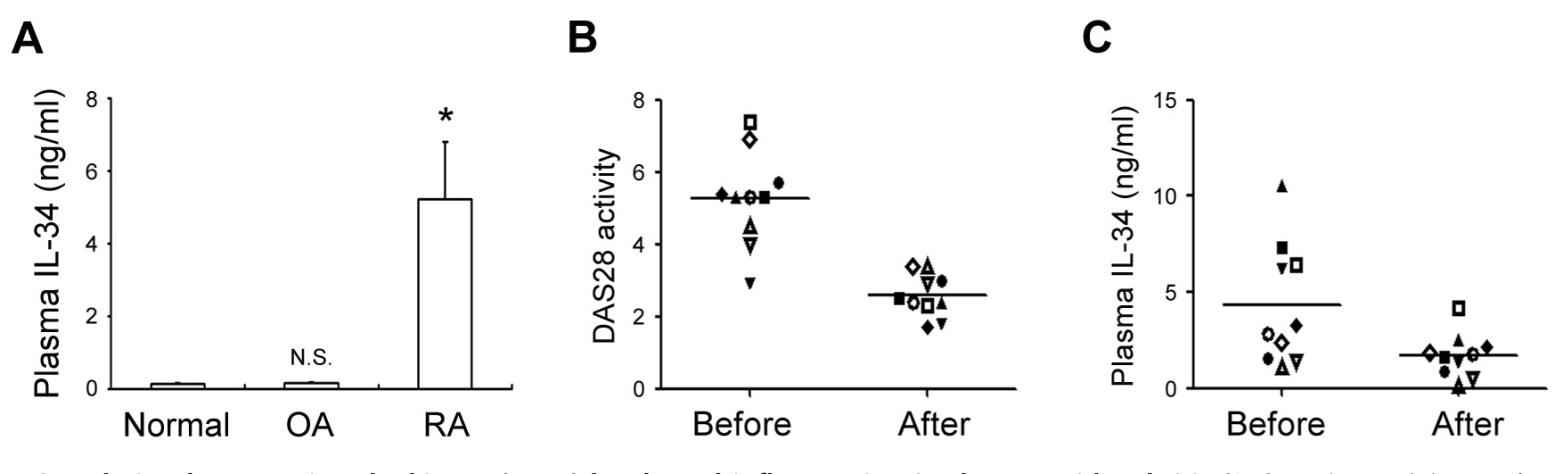
**Correlation between interleukin-34 (IL-34) levels and inflammation in rheumatoid arthritis (RA) patients**. (**A**) IL-34 levels were determined in plasma of healthy controls (normal), OA or RA patients by n enzyme-linked immunosorbent assay (ELISA) (*P < 0.05, N.S., not significant, compared to normal). (**B**) and (**C**) Plasma was collected from individual RA patients (*n *= 10) and the same patients were administrated disease-modifying anti-rheumatic drugs (DMARDs) and followed-up for 1 year. Disease Activity Score 28 (DAS28) activity (**A**) and IL-34 levels (ELISA) (**B**) were determined for each patient in plasma samples collected before and after administration of DMARDs. Symbols indicate individual data points; horizontal bars show group means (*P *< 0.01, after versus before DMARDs administration).

Given that IL-34 is up-regulated in inflamed synovium and in RA FLS in response to TNFα, (Figure 2) the levels of IL-34 may reflect the pathogenesis of inflammation in RA patients. To explore this correlation, we performed a comparative analysis of plasma IL-34 levels in RA patients (*n *= 10) using ELISAs. The patients with RA had higher plasma IL-34 levels than normal or OA controls (Figure 4A). One group of patients with rheumatoid arthritis, defined by a plasma DAS28 score at baseline of 2.9 to 7.4, had undergone a 1-year follow-up treatment with DMARDs, including methotrexate, sulfasalazine, leflunomide, minocycline, and/or hydroxychloroquine. Patients in this group (*n *= 10) showed an apparent decrease in DAS28 score of approximately 50% (to 1.7 to 3.4) after taking DMARDs (Figure 4B). Importantly, high levels of soluble IL-34 (6.573 ± 2.003 ng/ml) were detected in plasma from RA patients before DMARDs treatment (Figure 4C). However, a significant decrease (*P *< 0.01) in plasma IL-34 levels was observed after taking DMARDs in this group (Figure 4C). These data suggest that the plasma level of IL-34 correlates with the level of inflammation in RA patients.

### Effect of IL-34 on PBMCs migration and subsequent OC formation

**Figure 5:**
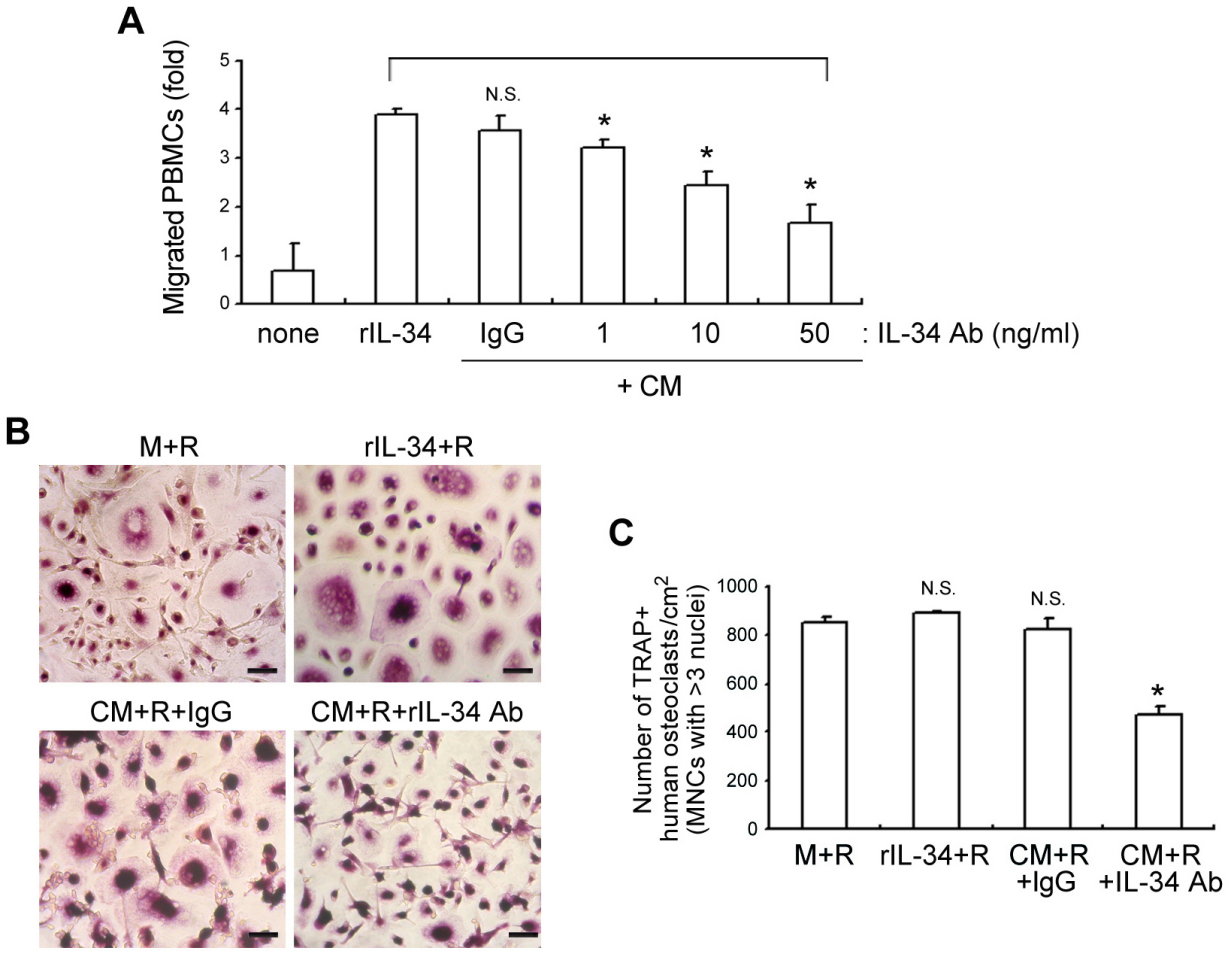
**Effect of interleukin-34 (IL-34) on human peripheral blood mononuclear cells (PBMCs) migration and subsequent osteoclast (OC) formation**. **(A**) Involvement of IL-34 in the migration of human PBMCs. Recombinant IL-34 (100 ng/ml) or CM (conditioned media) from rheumatoid arthritis (RA) fibroblast-like synovial cell (FLS) cultures with mouse immunoglobulin G (IgG) (50 ng/ml) or IL-34 antibody (Ab) (0, 1, 10, and 50 ng/ml) was added to the lower compartments of a transwell system, and PBMCs (1.2 × 10^6^) were added to the upper chamber of each transwell, Cells were allowed to migrate for six hours, and the number of PBMCs in the lower chamber was counted. Bars indicate the mean and standard deviation (SD) of the fold-change of PBMC migration relative to that of rIL-34 treatment (*P < 0.05, N.S., not significant, compared to PMBCs treated with rIL-34). (**B**) and (**C**) Involvement of IL-34 in osteoclast (OC) differentiation and activation. Isolated human PBMCs were cultured with macrophage colony-stimulating factor (M-CSF) (100 ng/ml) or IL-34 (100 ng/ml) plus receptor activator of nuclear factor kappa-B ligand (RANKL) (100 ng/ml) as a positive control or CM plus RANKL (100 ng/ml) in the presence of mouse IgG (50 ng/ml) or IL-34 Ab (50 ng/ml) for 12 to 14 days. Human osteoclasts showing tartrate resistant acid phosphatase (TRAP) positive (TRAP^+^) multinucleated cells (MNCs) (> 3 nuclei) on the same surface area were identified by TRAP staining (**B**) and counted under a light microscope (**C**). Scale bars = 50 μm. Bars indicate the mean and standard deviation (SD) of triplicate samples (*P < 0.05, N.S., not significant, compared to PBMCs cultured with M + R). rIL-34, recombinant IL-34; M, M-CSF; R, RANKL; none, no treatment; CM, conditioned media; IgG, isotype control; IL-34 Ab, anti-IL-34 blocking antibody.

Having shown that RA FLS produce IL-34 (Figure 2A and 3), we thus questioned whether IL-34 produced by RA FLS has functional activities to induce chemotactic migration of OCPs and to subsequently induce osteoclastogenesis as a substitute for M-CSF in RANKL-induced osteoclastogenesis. Human PBMCs as OCPs [27] in the upper chamber and CM from TNFα-treated RA FLS or recombinant IL-34 (positive control) in the lower chamber were incubated. Approximately 7.5 × 10^5 ^human PBMCs migrated in the presence of recombinant IL-34; a similar level of cell migration was observed in the presence of CM, which contains soluble IL-34 secreted from RA FLS (Figure 5A). However, addition of a blocking Ab against IL-34 to CM reduced the number of migrated human mononuclear cells in a dose-dependent manner.

To further assess the effects of IL-34 produced by RA FLS on RANKL-induced OC differentiation, we cultured isolated human PBMCs with RANKL in the presence or absence of M-CSF, IL-34, CM, or an anti-IL-34 Ab for 12 to 14 days. M-CSF plus RANKL treatment induced OC differentiation (851.5 ± 17.7; Figure 5B). IL-34 or CM from RA FLS cultures containing IL-34 supported RANKL-induced OC formation to an extent similar to that of M-CSF. The addition of anti-IL-34 antibody to block functional IL-34 in CM (CM + R + IL-34 Ab) significantly reduced OC formation (472.4 ± 39; *P *= 0.042) (Figure 5C). These data demonstrate that IL-34 secreted by RA FLS is functionally active enough to stimulate the chemotactic migration of OCPs and subsequent OC formation.

## Discussion

Although RANKL is absolutely required for OC differentiation *in vivo *[28,29], several cytokines, including vascular endothelial growth factor and hepatocyte growth factor, can be substituted for M-CSF in promoting osteoclastogenesis [30-32]. The inflamed synovium of RA patients is the major source of overproduction of cytokines, and these cytokines may contribute to osteoclastic activation [33]; thus, understanding the specific factors capable of replicating the effect of M-CSF is of considerable research interest.

Inflammatory cytokines, including IL-1β, IL-17 and TNFα, are elevated in the SF of RA patients [34] and are known to stimulate osteoclastogenesis through induction of M-CSF [35-37]. In this study, we demonstrated that both IL-34 mRNA and protein expression were up-regulated by TNFα to a greater extent in RA FLS than in OA FLS, a pattern distinct from that of M-CSF expression (Figure 2). Thus, this is the first report describing the stimulation of IL-34 expression by TNFα in FLS of RA patients, suggesting that RA FLS are much more susceptible to the production of IL-34 than M-CSF in response to TNFα. Further study implies a discrete role of IL-34 in the pathological inflammatory setting of RA.

A recent report demonstrated that TNFα induces IL-34 mRNA via JNK and NF-κB, but not the p38 pathway, in osteoblasts [38]. NF-κB, in particular, is responsible for TNFα-induced M-CSF expression in primary osteoblast cells [39]. In our experiments, TNFα-mediated induction of IL-34 expression was inhibited by an NF-κB inhibitor, indicating that IL-34 expression is regulated, at least in part, by NF-κB. Moreover, JNK inhibition modestly decreased the elevation of IL-34 expression, indicating that JNK may also be involved in IL-34 expression in FLS of RA patients (Figure 3D).

Several combinations of DMARDs used for the treatment of arthritis conditions [40], such as RA or psoriatic arthritis (PsA), have been demonstrated to abrogate inflammation [41]. IL-34 was detected in plasma samples of RA patients and it was effectively reduced by DMARDs treatment in RA patients (Figure 4A). Our data provide an initial indication of the importance of the clinical correlation between plasma IL-34 levels and inflammatory responses in RA patients, suggesting the significance of IL-34 as a plasma factor in RA patients representing inflammatory arthritis in the pathogenesis of RA. In our study population, individual variations in combination therapy were not controlled; thus, additional clinical studies with a larger number of patients divided into subgroups is needed to validate the clinical implications of elevated IL-34 in the pathogenesis of RA.

IL-34, like M-CSF, has been shown to promote osteoclastogenesis in combination with RANKL [14,18] and induce bone resorption activity [18]. We also found that IL-34 produced from TNFα-stimulated RA-FLS promoted chemotactic migration of human PBMCs and RANKL-induced osteoclastogenesis (Figure 5), demonstrating a biologically active function of IL-34. Therefore, TNFα-stimulated up-regulation of IL-34 in RA FLS may play a pathological role in RA, possibly at the site of inflammatory erosive bone resorption and local bone destruction. In PsA, an inflammatory joint disease characterized by extensive bone resorption, OCPs arise from TNFα-activated PBMCs that migrate to the inflamed synovium and subchondral bone, where they are exposed to unopposed RANKL and TNFα [42]. This leads to osteoclastogenesis at the erosion front and in subchondral bone, resulting in a bidirectional assault on psoriatic bone [42]. A recent report showed that M-CSF levels are not elevated in SF from patients with PsA to the same extent as in RA patients [12]. Accordingly, IL-34 may replace this M-CSF function and contribute to the pathogenesis of PsA; however, the role of IL-34 in PsA remains to be investigated.

Genetic ablation of M-CSF in *op/op *(M-CSF-deficient) mice and inhibition of M-CSF by administration of anti-M-CSF mAbs exerts protective effects in a number of inflammatory and/or autoimmune conditions [43,44]. Moreover, blocking c-Fms inhibits macrophage infiltration, TNFα production by macrophages, and OC formation and activation in several mouse arthritis models [12,45], and reduces TNFα-induced inflammatory arthritis [11], suggesting a therapeutic benefit in RA treatment. The increase in IL-34 in RA reported here supports the concept that IL-34 regulates OCP migration and subsequent OC differentiation, suggesting the possibility that IL-34 can functionally substitute for M-CSF and is involved in the RA disease process elicited by TNFα. It is important to note in this context that elevated IL-34 in RA SF can activate c-Fms, even in the absence of M-CSF. Thus, inhibiting c-Fms may be a more promising strategy than targeting M-CSF in the treatment of RA where the disease pathogenesis involves TNFα.

## Conclusions

We propose that IL-34 is elevated in RA SF and is produced by RA FLS. IL-34 expression is enhanced by TNFα through the NF-κB and JNK pathway and is a biologically active stimulator of OC differentiation that exerts synergistic effects with RANKL. Further investigations to define the pathological implications of this cytokine in osteolysis *in vivo *are warranted.

## Abbreviations

Ab: antibody; BSA: bovine serum albumin; CM: conditioned media; DAS28: disease activity score 28; DMARDs: disease-modifying antirheumatic drugs; ERK: extracellular signal-regulated protein kinase; FLS: fibroblast-like synovial cells; HRP: horseradish peroxidase; IL-34: interleukin-34; JNK: c-Jun N-terminal kinase; MAPK: mitogen-activated protein kinase; M-CSF: macrophage colony-stimulating factor; NF-κB: nuclear factor kappa B; OA: osteoarthritis; OC: osteoclast; OCPs: osteoclast precursors; PBS: phosphate-buffered saline; PDTC: pyrrolidine dithiocarbamate; PsA: psoriatic arthritis; PBMCs: peripheral blood mononuclear cells; qRT-PCR: quantitative reverse transcriptase -polymerase chain reaction; RA: rheumatoid arthritis; RANKL: receptor activator of NF-κB ligand; RT-PCR: reverse transcriptase-polymerase chain reaction; SF: synovial fluid; TNFα: tumor necrosis factor alpha; TRAP: tartrate-resistant acid phosphatase.

## Competing interests

The authors declare that they have no competing interests.

## Authors' contributions

SH, BC, and SK performed experiments, analyzed data, and wrote the manuscript. JC and YC performed FLS and PBMCs isolations. DS performed immunohistochemistry. WHR was involved in study design and data analysis. YK and CL, as clinical supervisors, provided RA SF and synovial tissues. MS performed statistical analysis on the patients' plasma samples. EC designed the study, contributed to data analysis, and wrote the manuscript. All authors have read and approved the final manuscript.

## Supplementary Material

Additional file 1 **Characteristics of patients with rheumatoid arthritis (RA)**. All patients were 30 years or older at the time of RA diagnosis and their mean ± SD age was 53.3 ± 16.0 years. All patients were positive for rheumatoid factor (RF) and anti-cyclic citrullinated peptide (CCP) antibody and had a diagnosis of RA with a median baseline Disease Activity Score 28 (DAS28) of 5.27 (range 2.9 to 7.4), a median 1 year DAS28 of 2.58 (1.7 to 3.4), a baseline median erythrocyte sedimentation rate of 69.6 (28 to 120), and a median baseline C-reactive protein (CRP) of 1.05 (0.1 to 2.04). With regard to medications used for the treatment of RA, patients were treated with prednisolone and disease-modifying antirheumatic drugs (DMARDs) including methotrexate, sulfasalazine, leflunomide, FK506, and/or hydroxychloroquine. SD, standard deviation. Click here for file
