# Supplementary material for: Interleukin-34 produced by human fibroblast-like synovial cells in rheumatoid arthritis supports osteoclastogenesis
Source: Arthritis Res Ther. 2012 Jan 20;14(1):R14. doi: 10.1186/ar3693 (PMC3392804; doi:10.1186/ar3693)
Supplement: Additional file 1 — Characteristics of patients with rheumatoid arthritis (RA). All patients were 30 years or older at the time of RA diagnosis and their mean ± SD age was 53.3 ± 16.0 years. All patients were positive for rheumatoid factor (RF) and anti-cyclic citrullinated peptide (CCP) antibody and had a diagnosis of RA with a median baseline Disease Activity Score 28 (DAS28) of 5.27 (range 2.9 to 7.4), a median 1 year DAS28 of 2.58 (1.7 to 3.4), a baseline median erythrocyte sedimentation rate of 69.6 (28 to 120), and a median baseline C-reactive protein (CRP) of 1.05 (0.1 to 2.04). With regard to medications used for the treatment of RA, patients were treated with prednisolone and disease-modifying antirheumatic drugs (DMARDs) including methotrexate, sulfasalazine, leflunomide, FK506, and/or hydroxychloroquine. SD, standard deviation. [file ar3693-S1.DOC]

Characteristics of patients with rheumatoid arthritis (RA).

| **Pateint No.** | **Baseline**  **DAS28** | **1Yr**  **DAS28** | **Gender^1^** | **Age^2^** | **RF^3^** | **CCP^5^** | **Baseline**  **ESR**  **(mm/hr)** | **Baseline**  **CRP (mg/dl)** | **PD^6^**  **(mg/**  **Year)** | **Methotrexate** | **SALA^7^** | **HCQ^8^** | **Leflunomide** | **FK506** |
| --- | --- | --- | --- | --- | --- | --- | --- | --- | --- | --- | --- | --- | --- | --- |
| 1 | 5.4 | 1.7 | F | 60 | +^4^ | +^4^ | 99 | 0.98 | 2690 | +^9^ | -^9^ | -^9^ | -^9^ | +^9^ |
| 2 | 5.3 | 2.4 | F | 58 | + | + | 75 | 1.47 | 2570 | + | - | +^9^ | +^9^ | -^9^ |
| 3 | 5.7 | 3 | F | 30 | + | + | 57 | 0.3 | 4060 | + | - | - | - | - |
| 4 | 5.3 | 2.5 | F | 49 | + | + | 83 | 0.58 | 1385 | + | - | + | - | - |
| 5 | 5.3 | 2.4 | F | 78 | + | + | 59 | 1.83 | 1343 | + | - | + | - | - |
| 6 | 2.9 | 1.8 | F | 47 | + | + | 81 | 0.6 | 1458 | + | - | + | - | - |
| 7 | 4.5 | 3.4 | F | 43 | + | + | 28 | 0.1 | 878 | + | + | + | - | - |
| 8 | 7.4 | 2.3 | M | 48 | + | + | 46 | 2.04 | 4113 | + | - | + | + | - |
| 9 | 4 | 2.9 | F | 62 | -^4^ | + | 48 | 0.93 | 100 | + | - | - | - | - |
| 10 | 6.9 | 3.4 | F | 58 | + | + | 120 | 1.63 | 2190 | + | - | + | - | - |

All patients were 30 years or older at the time of RA diagnosis and their mean±SD age was 53.3±16.0 years. All patients were positive for rheumatoid factor (RF) and anti-cyclic citrullinated peptide (CCP) antibody and had a diagnosis of RA with a median baseline disease activity score (DAS) 28 of 5.27 (range 2.9-7.4), a median 1 year DAS28 of 2.58 (1.7-3.4), a baseline median erythrocyte sedimentation rate of 69.6 (28-120), and a median baseline C-reactive protein (CRP) of 1.05 (0.1-2.04). With regard to medications used for the treatment of RA, patients were treated with prednisolone and disease-modifying antirheumatic drugs (DMARDs) including methotrexate, sulfasalazine, leflunomide, FK506, and/or hydroxychloroquine. ^1^Gender: F, female; M, male, ^2^Age: age diagnosed with RA, ^3^RF: Rheumatoid factor, ^4^+, positive; -, negative, ^5^CCP: anti-CCP antibody, ^6^PD: cumulative dose of Prednisolone for 1 year, ^7^SALA: Sufasalazine, ^8^HCQ: Hydroxychloroquine, ^9^+; applied, -; not applied
